# The Regulation of ZAR1 on Apoptosis and Mitophagy in Ovarian Granular Cells and Primary Ovarian Insufficiency (POI) Mice

**DOI:** 10.1007/s43032-025-01857-z

**Published:** 2025-04-11

**Authors:** Xiaodong Zhao, Bin Mao, Jianwen Wang, Huabin Wang, Xiaoling Ma, Kehu Yang, Yongxiu Yang

**Affiliations:** 1 The First School of Clinical Medicine, Lanzhou University, Lanzhou, China; 2 The First Hospital of Lanzhou University, Lanzhou, China; 3 The School of Basic Medical Sciences, Lanzhou University, Lanzhou, China

**Keywords:** Apoptosis, Autophagy, Ovarian granulosa cell, Primary ovarian insufficiency, ZAR1

## Abstract

Primary ovarian insufficiency (POI) has become a serious problem causing infertility and endocrine disorders in women of child-bearing age. There is an urgent demand for novel drugs or targets to address the apoptosis, autophagy and mitochondria damage associated with ovarian aging. This study focused on the regulation of zygote arrest 1 (ZAR1) in ovarian function and its potential role in POI. We collected clinical samples, established POI cell and mouse models using 4-vinylcyclohexene diepoxide (VCD), and investigated the effects of ZAR1 in KGN cells and POI mice. We found that ZAR1 expression was negatively associated with follicle-stimulating hormone (FSH) in POI women. ZAR1 overexpression inhibited apoptosis activation, cell cycle arrest and mitophagy, but the protection effects can be blocked by autophagy inhibitor. Mice with lower expression of ZAR1 exhibited more severe ovarian damages. These findings indicated that ZAR1 is a novel target for prevention and treatment of ovarian aging.

**Supplementary Information:**

The online version contains supplementary material available at 10.1007/s43032-025-01857-z.

## Introduction

The ovarian follicular maturation or folliculogenesis is an intricate and long process. Small primordial follicles gradually develop into large ovulatory follicles and release from the surface of the ovary, collected by the fallopian tube, transport to the uterus, and fertilized or discarded [1]. Ovarian reserve refers to the number of oocytes in the ovary and determines the female reproductive lifespan [2]. The acceleration of follicle loss and the reduction in oocyte quality can cause a series of reproductive problems in women and eventually lead to infertility.

Primary ovarian insufficiency (POI) is a reproductive disorder affecting approximately 1% of women under 40 years old and 0.1% of women under 30 years old [3]. POI is characterized by elevated gonadotropin levels, low estrogen levels, amenorrhea, and insufficient mature follicles [4, 5]. It significantly impacts fertility and causes endocrine dysfunction in women of reproductive age. Women with POI have lower clinical pregnancy rates and embryo implantation rates, companies with an increased risk of recurrent pregnancy loss [6–8].

The infertility caused by POI is often due to insufficient primordial follicle reserve, accelerated follicular atresia, changes in dominant follicle recruitment, follicular maturation disorders, and low response to gonadotropin stimulation [9]. The etiologies of POI are related to various factors, including hereditary factor, iatrogenic injury, autoimmune and endocrine diseases, environmentally disruptive chemicals, mitochondrial dysfunction and infection [10]. Among these, about 20-25% of cases are associated with genetic abnormality [11]. Known hereditary factors in POI include autosomal and X chromosome abnormalities and variants of candidate genes, such as growth differentiation factor 9 *(GDF9)*, bone morphogenetic protein 15 *(BMP15)*, minichromosome maintenance complex component 8 *(MCM8)*, X-ray repair cross complementing protein4 *(XRCC4)* and fragile X mental retardation 1 *(FMR1)* [12]. Given the familial aggregation of POI, further study on its molecular mechanisms is warranted.

In mammals, transcription stops at the last stage of oocyte maturation and resumes after fertilization. During this time, proteins synthesis in oocytes depends solely on stored mRNA highlighting the significance of mRNA storage in mature haploid ova [13]. Zygote arrest 1 (*ZAR1*), a RNA-binding protein, is essential for the full-grown mouse oocytes [14]. *ZAR1* is an ovary-specific maternal gene highly conserved in vertebrates [15]. The human *ZAR1* is located on chromosome 4 (4p11) and is companied by a large 1.5 kb CpG island [16]. Besides the ovary, *ZAR1* is also expressed in heart, brain, lung, kidney, testicle and muscle [17–19]. Mice *ZAR1* show high homology with human *ZAR1*. Previous study has reported that knocking out *ZAR1* in mice is non-lethal but cause female infertility [20]. Although ovarian development, oogenesis, and early stages of fertilization are not disrupted, embryos from *ZAR1* knockout mice exhibit disordered cell division and can only develop to early stages, highlighting the significance of *ZAR1* in early embryo development [21]. The necessity of *ZAR1/2* on oocyte meiotic maturation was also reported for oocyte maturation retardation occurred in double knockout female mice [14].

Previous studies have shown the importance of ZAR1 in oocyte development, its role in POI has not been extensively explored. Given the importance of ZAR1 in folliculogenesis and the impact of POI, we investigated the potential association between ZAR1 and POI. We focused on ovarian granulosa cells and POI mice to explore the molecular regulatory mechanisms of ZAR1.

## Methods

### Clinical Samples Collection

The study recruited women who accepted oocyte pickup in the Lanzhou University First Affiliated Hospital. The study population comprised the control group (*n* = 23) and the POI group (*n* = 25). Inclusion criteria included women aged 18–40 with regular menstrual cycles and no history of ovarian surgery. Exclusion criteria included a history of chemotherapy, radiation therapy, or chronic systemic diseases (e.g., diabetes, autoimmune disorders). Informed consent was gained from all participants. The study was approved by the Ethics Committee of the Lanzhou University First Affiliated Hospital.

Follicular fluid samples were centrifuged at 3000 rpm for 10 min and the upper fluid was stored at -80 ℃.

### Cell Culture

KGN cells, a human granulosa cell line, were purchased from Procell (Wuhan, China). The KGN cells were cultured in DMEM medium containing 10% FBS and 1% penicillin-streptomycin solution, maintained in cell culture incubator at 37 ℃ with 5% CO_2_.

### Mice Treatment

Female C57BL/6 mice (6–8 weeks old) were purchased from Lanzhou Veterinary Research Institute and housed under specific pathogen-free conditions with a temperature of 22 ℃ ± 1 ℃ and humidity of 60% ± 10%. The mice had free access to food and water during rearing. All procedures were approved by the Animal Ethics Committee of the First Hospital of Lanzhou University.

The mice were randomly divided into control group (*n* = 10) and POI group (*n* = 10). The mice of POI group were treated with VCD (160 mg/kg) by intraperitoneal injection, and the mice of control group were treated with the same volume of corn oil. The VCD dose (160 mg/kg) was selected based on previous studies demonstrating its efficacy in inducing ovarian insufficiency in mice [22, 23]. The injection was continued for 20 days and then the mice were executed under anesthetic.

### Realtime Quantitative Polymerase Chain Reaction (PCR)

Total RNA was extracted by TRIzol reagent (Invitrogen Life Technologies, USA) and reverse transcribed to cDNA by FastKing gDNA Dispelling RT SuperMix kit (TIANGEN BIOTECH, Beijing) following the instruction. The SuperReal PreMix Plus (SYBR Green) kit was used for quantitative PCR amplification. *GAPDH* was selected as the reference gene.

### Enzyme-Linked Immunosorbent Assay (ELISA)

Follicular fluid and mouse serum were collected, centrifuged, and stored at -80 ℃. The levels of ZAR1, FSH, catalase (CAT), glutathione peroxidase (GPX), malondialdehyde (MDA), and superoxide dismutase (SOD) were measured using ELISA kits (FINGHUISHENGWU, China) according to standard protocols.

### Cell Transfection

The overexpression plasmid of *ZAR1* was obtained from HANBIO (Shanghai, China). LiPOIectamine™ 3000 Reagent kit (Thermo Fisher Scientific Company, USA) was used for transfection. The KGN cells were seeded into 12-well plates (1 × 10^5^cell) and preincubate for 24 h. Each well was washed by PBS. The 2 µL transfection reagent, 5 µL (0.2 µg/µL) plasmid and 50 µL Opti-MEM were mixed and incubated for 15 min at room temperature. Finally, the mixture was added into KGN cells and cultured for 48 h.

### Cell Viability Assay

Cell Counting Kit-8 (CCK8; Coolaber, China) was used to measure cell viability. The KGN cells (5 × 10^3^) were seeded in the 96-well plates and treated following experiment scheme. Then we added 10 µL CCK8 and 90 µL serum-free medium to each well. The samples were incubated for 3 h at 37 ℃ and detected with microplate reader (TECAN, Switzerland).

### Western Blotting

The protein was extracted from KGN cells and ovary of mice. Proteins (30 µg) were separated by electrophoresis in 10% SDS polyacrylamide gels and then transferred to polyvinylidene fluoride (PVDF) membrane. After blocked with 5% non-fat milk at room temperature for 2 h. Then, the membranes were incubated with primary antibody at 4 ℃ overnight. The primary antibodies including Anti-beta-Actin (1:10000, Bioss), Anti-Bax (1:1000, Bioss), Anti-Bcl-2 (1:1000, Bioss), Anti-caspase-3(1:1000, Bioss), Anti-Cyclin C (1:1000, Bioss), Anti-Cyclin D1 (1:1000, Bioss), Anti-Cyclin E (1:1500, Bioss), Anti-CDK2 (1:800, Bioss), Anti-LC3 (1:1000, Bioss), Anti-p62 (1:2000), Anti-ZAR1 (1:1000, Bioss). Then the membranes were incubated with HRP-conjugated anti-rabbit IgG for 2 h. Finally, the enhanced chemiluminescence (ECL) detection kit (BioRad, USA) was used for proteins detection. The blots were visualized with ChemiDoc XRS + imaging system (Bio-Rad Laboratories, USA) and analyzed with ImageJ.

### Apoptosis Detection

Annexin-V PE/7-AAD apoptosis kit (4 A BIOTECH, China) was used for apoptosis detection. KGN cells were seeded into 6-well plate and incubated for 24 h. Then the cells were harvested with trypsin and resuspended with 500 µL 1x binding buffer, then 5 µL PE and 10uL 7-AAD was added into the mixture. Finally, detected the level of apoptosis with flow cytometry (BD, USA).

### Cell Cycle Detection

Cell cycle detection kit (4 A BIOTECH, China) was used to detect the changes of cell cycle. KGN cells were seeded into 6-well plate and incubated for 24 h. Then the cells were harvested with trypsin and fixed with 500 µL 70% ethyl alcohol for 2 h. After centrifugation, the 70% ethyl alcohol was removed. Then the sediment was resuspended with 100 µL RNase A and dyed with 400 µL PI for 30 min. Finally, the cell cycle changes were detected with flow cytometry (BD, USA).

### ATP Detection

The ATP content detection kit (Solarbio, China) was used for ATP detection. KGN cells (10^5^) were sonicated for 1 min and centrifuged for 10 min at 4 ℃. The supernatant was mixed with 500µL chloroform and centrifuged for 3 min, then extracted the supernatant to detect the ATP content according to the instruction.

### Mitochondrial Membrane Potential Detection

The mitochondrial membrane potential detection kit (Beyotime, China) was used for detection of mitochondria damage. First, we seeded 10^5^ KGN cells in the 6-well plate and cultured for 24 h. The 1000x TMRE was diluted to 1x TMRE with buffer solution and added 1mL to each well, then incubated with cells for 30 min at 37 ℃. After washing the cells with serum-free medium we assess the mitochondrial membrane potential level by flow cytometry (BD, USA).

### Reactive Oxygen Species (ROS) Detection

The ROS detection kit (Solarbio, China) was used for the assessment of level of ROS. First, we seeded 10^5^ cells in the 6-well plate and cultured for 24 h. Then we diluted DHE dye with serum-free medium (1:1000). The cells were washed with PBS solution and then dyed with the diluted DHE dye for 30 min at 37 ℃. After washing the cells with serum-free medium thrice, we observed the fluorescence intensity under the fluorescence microscope (Olympus, Japan).

### Ad-mCherry-GFP-LC3B Transfection

A total of 10^5^ cells were seeded in 12-well plates and cultured for 24 h. Cells were grown on 12-well plates and reached 20–30% confluence at the time of transfection. After washed with PBS, cells were transfected with mCherryGFP-LC3B adenovirus in serum-free medium containing polybrene (5 µg/mL) for 24 h at 37 ℃. Following designed treatment, autophagosome and autophagy flux were observed under fluorescence microscopy (Olympus, Japan).

### H&E Staining

The ovaries of mice were fixed in 10% formalin for 24 h, embedded in paraffin and cut into 5 μm thick. After routine deparaffinization, the tissues were stained with hematoxylin for 5 min, flushed for 3 min, and then stained with eosin for 1 min. Following dehydration, the sections were sealed by neutral gum and observed by microscope. Follicle classification was performed according to the Pederson standard [24, 25].

### Immunohistochemistry (IHC)

After slicing, drying, hydration and antigen retrieval, the ovary samples were incubated with peroxidase blocker for 30 min at room temperature. Then incubated samples with primary antibodies at 4 ℃ overnight. After rewarming for 1 h, added response enhancement solution to the samples. Then incubated samples with goat-anti-rabbit IgG for 20 min. After DAB dyeing, hematoxylin dyeing and regular dehydration. The samples were sealed and observed under the microscope.

### Statistical Analysis

Spearman correlation analysis was performed for the relationship between the level of FSH and the level of ZAR1 in women with or without POI. Comparisons between two groups were performed with T test. Comparisons among three or more groups were performed with one-way analysis of variance (ANOVA). *P* < 0.05 was considered as statistically significant. All data were analyzed by SPSS 25.0. All experiments were executed at least three times.

## Results

### The Expression of ZAR1 in Women with POI was Decreased

**Fig. 1:**
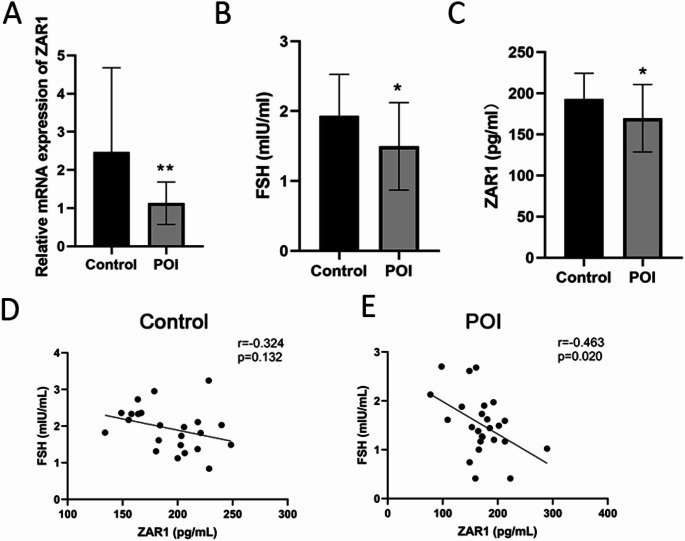
Expression of ZAR1 in women with premature ovarian insufficiency (POI). **A**. The expression level of ZAR1 mRNA in human follicular fluid in each group (control group *n*=23, POI group *n*=25). **B**. The level of follicle-stimulating hormone (FSH) in human follicular fluid in each group (control group *n*=23, POI group *n*=25). **C**. The level of ZAR1 in human follicular fluid in each group (control group *n*=23, POI group *n*=25). **D**. In the control group, Spearman correlation analysis was performed between FSH levels and ZAR1 levels. **E**. In the POI group, Spearman correlation analysis was performed between FSH levels and ZAR1 levels. *P<0.05 indicates a significant difference between the two groups

As shown in Fig. 1A, the mRNA expression of *ZAR1* was significantly decreased in women with POI. ELISA results showed that the FSH and ZAR1 levels were significantly decreased in women with POI (Fig. 1B, C). Then we conducted correlation analysis between ZAR1 and FSH in women with or without POI. A significant negative correlation was observed between ZAR1 expression and FSH levels in POI patients (*r* = -0.463, *p* < 0.05) (Fig. 1D).

### ZAR1 Overexpression Inhibits Apoptosis and Cell Cycle Arrest

**Fig. 2:**
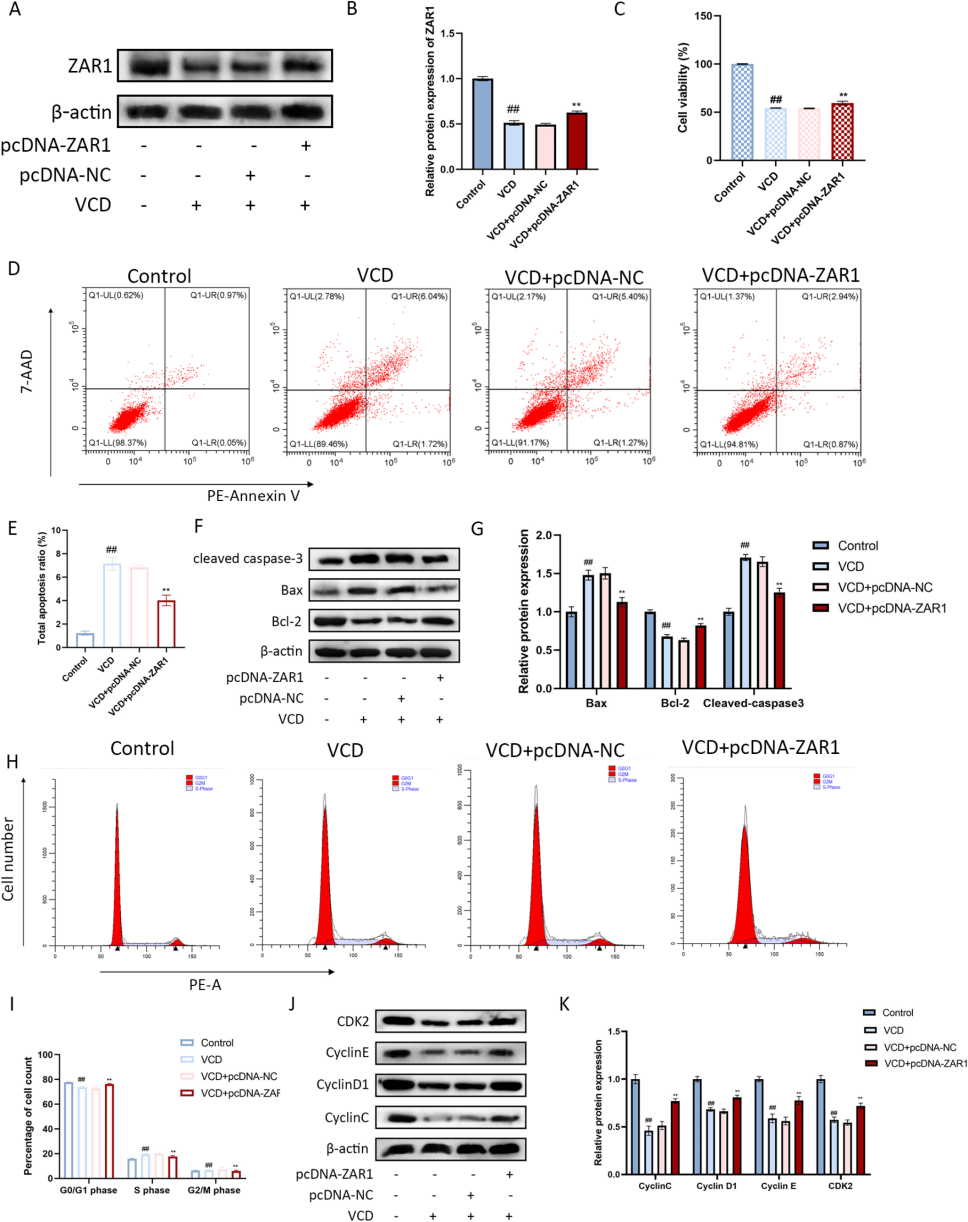
Regulation of ZAR1 on apoptosis and cell cycle in KGN cells. **A**. ZAR1 overexpression in KGN cells. **B-C**. The cell viability of KGN cells after ZAR1 overexpression. **D-E**. The apoptosis level of KGN cells after ZAR1 expression. **F-G**. The protein expression of Bax, Bcl-2 and cleaved caspase-3 after ZAR1 expression. **H-I**. The changes of cell cycle after ZAR1 expression. **J-K**. The protein expression of Cyclin C, Cyclin D1, Cyclin E and CDK2 after ZAR1 overexpression. ^#^*P*<0.05, ^##^*P*<0.01 vs. control group, ^*^P<0.05, ^**^P<0.01 vs. VCD+pcDNA-NC group

To study the biological function of ZAR1, we overexpressed ZAR1 in KGN cells (Fig. 2A, B). As shown in Fig. 2C, VCD treatment decreased cell viability, while ZAR1 overexpression significantly enhanced cell viability in VCD-treated KGN cells. The total apoptosis ratio was significantly increased in VCD-treated KGN cells, while ZAR1 overexpression reduced apoptosis levels (Fig. 2D, E). Western blotting revealed decreased expression of pro-apoptotic proteins (Bax and cleaved caspase-3) and increased expression of anti-apoptotic protein (Bcl-2) in ZAR1-overexpressing cells (Fig. 2F, G).

We also detected the effects of ZAR1 on the cell cycle. KGN cells treated with VCD alone had a lower proportion of G0/G1 phase cell and higher proportion of S phase cells, but the tendency was reversed after overexpressed ZAR1 (Fig. 2H, I).

Cyclin C, Cyclin D1 and Cyclin E were only express in G1 phase, their protein expressions were dramatically decreased and significantly increased after ZAR1 expression (Fig. 2J, K). These results showed that ZAR1 can inhibit the apoptosis and alleviate the cell cycle arrest in KGN cells.

### ZAR1 Overexpression Inhibits Mitophagy

**Fig. 3:**
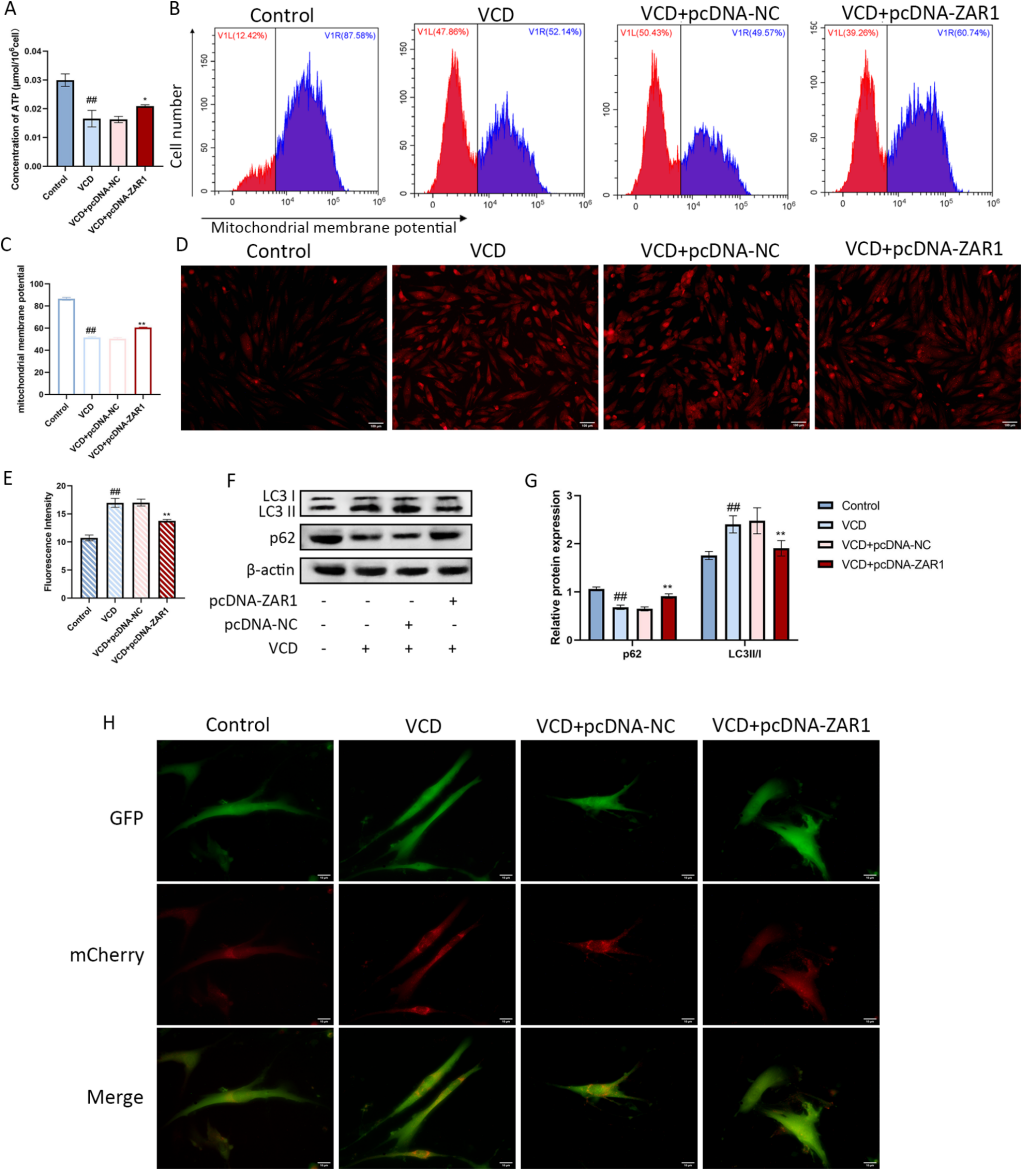
Regulation of ZAR1 on mitophagy. **A**. The concentration of ATP after ZAR1 overexpression. **B-C**. The change of mitochondrial membrane potential after ZAR1 overexpression. **D-E**. The level of ROS accumulation after ZAR1 overexpression. Scale bar=100 μm. **F-G**. The protein expression of LC3 and p62 after ZAR1 overexpression. **H**. Representative images of GFP and mCherry Fuorescent puncta in KGN cells transfected with mCherry-GFP-LC3B adenovirus for 24 h. Scale bar=10 μm. ^#^*P*<0.05, ^##^*P*<0.01 vs. control group, ^*^P<0.05, ^**^P<0.01 vs. VCD+pcDNA-NC group

KGN treated with VCD had a lower concentration of ATP. Although the concentration of ATP was still lower than the control group, it was significantly increased in the ZAR1 expression group (Fig. 3A). The flow cytometry also showed the lower mitochondrial membrane potential was increased after ZAR1 expression (Fig. 3B, C). Then we detected the ROS level in KGN cells. The red fluorescence means ROS accumulation. Compared with the vector group, ZAR1 overexpression group had weaker fluorescence intensity which means less ROS accumulation (Fig. 3D, E). These results showed that ZAR1 can relieve the mitochondria damages.

The detections on autophagy were also performed. In KGN cells treated with VCD, the expression of LC3 II/I was increased and the expression of p62 was decreased while ZAR1 overexpression against the autophagy activation (Fig. 3F, G). Figure 3H showed the effects of ZAR1 on autophagosomes. The red puncta indicate autophagosomes and autolysosomes, because green fluorescent protein (GFP) loses its fluorescence in an acidic environment while mCherry keeps its fluorescence. The yellow puncta indicate autophagosomes while the red puncta indicate autolysosomes. The VCD treatment caused autophagy flux activation while ZAR1 overexpression blocked the autophagy flux (Fig. 3H). These results showed that ZAR1 can inhibited the mitophagy activation in KGN cells.

### Baf-A1 Reverses the Effects of ZAR1 on Apoptosis and Cell Cycle

**Fig. 4:**
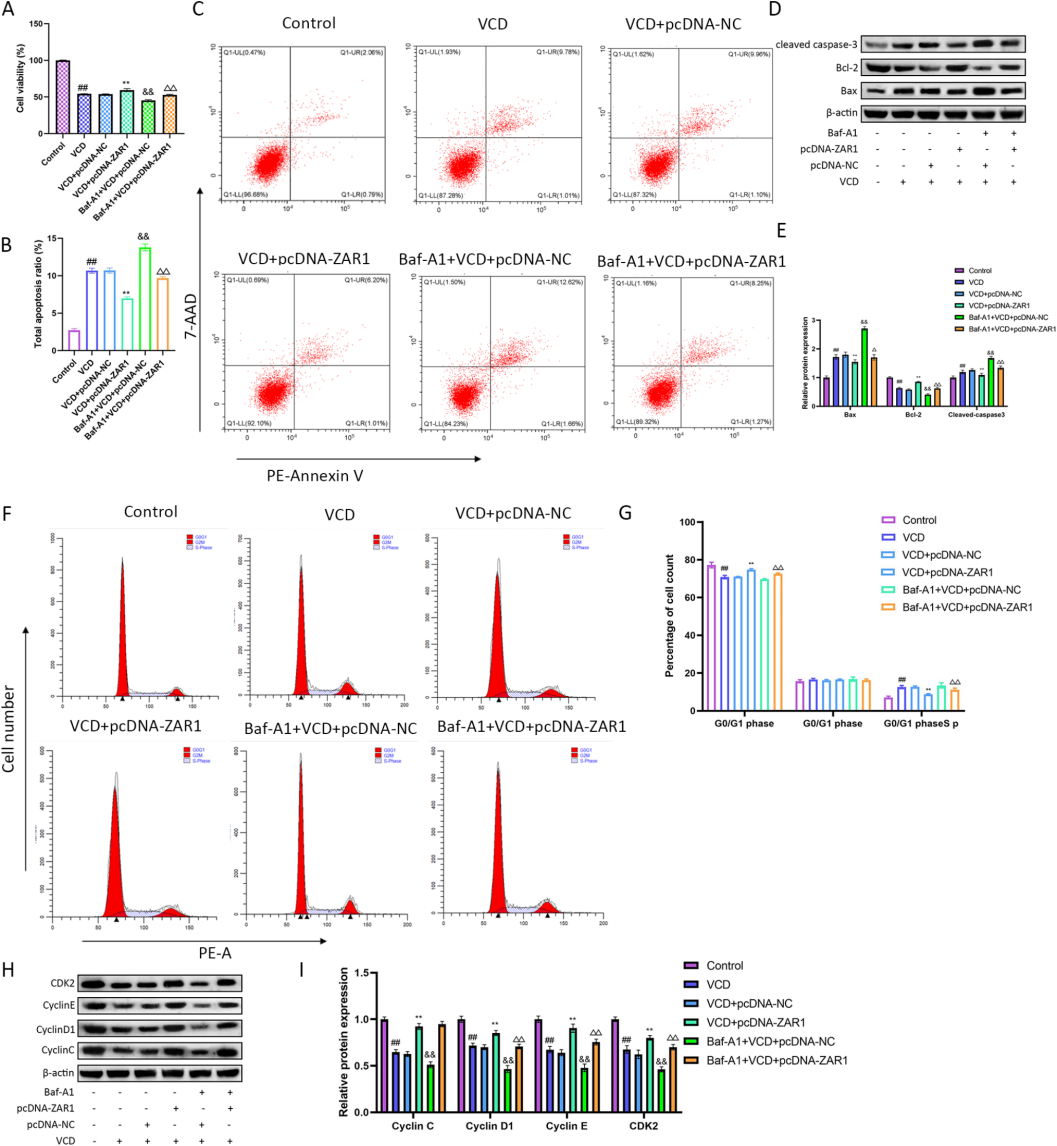
Baf-A1 reversed the regulation of ZAR1 on apoptosis and cell cycle. **A**. The cell viability after treated with Baf-A1 and ZAR1 overexpression. **B-C**. The apoptosis level of KGN cells after treated with Baf-A1 and ZAR1 overexpression. **D-E**. The protein expression of Bax, Bcl-2 and cleaved caspase-3 after treated with Baf-A1 and ZAR1 overexpression. **F-G**. The changes of cell cycle after treated with Baf-A1 and ZAR1 overexpression. **H-I**. The protein expression of Cyclin C, Cyclin D1, Cyclin E and CDK2 after treated with Baf-A1 and ZAR1 overexpression. ^#^*P*<0.05, ^##^*P*<0.01 vs. control group, ^*^P<0.05, ^**^P<0.01 vs. VCD+pcDNA-NC group, ^&^*P*<0.05, ^&&^*P*<0.01 vs. VCD+pcDNA-NC group, ^△^*P*<0.05, ^△△^*P*<0.01 vs. VCD+pcDNA-ZAR1group

To study whether the regulation of ZAR1 is mediated by autophagy, we used the autophagy inhibitor Baf-A1 in the experiment. Baf-A1 is a well-known inhibitor of autophagy that blocks the fusion of autophagosomes with lysosomes, thereby inhibiting autophagic flux [26, 27]. First, we observed that compared with ZAR1 overexpression group, the addition of Baf-A1 caused notable cell viability decrease (Fig. 4A). As shown in Fig. 4B and C, ZAR1 reduced the apoptosis level increase caused by VCD, while after Baf-A1 treatment, the apoptosis level elevated again. The expression of Bax, Bcl-2 and cleaved caspase-3 also supported that the alleviation of ZAR1 to apoptosis can be inhibited by autophagy inhibitor (Fig. 4D, E).

The detection on cell cycle showed that combined Baf-A1 and ZAR1 overexpression caused the proportion of G0/G1 phase cell increase and the proportion of G2/M phase cell decrease (Fig. 4G). Meanwhile, the expressions of Cyclin D1 and Cyclin E were notably down-regulated after combined Baf-A1 and ZAR1 overexpression (Fig. 4H, I).

### Baf-A1 Inhibits the Protective Effects of ZAR1 on Mitochondria

**Fig. 5:**
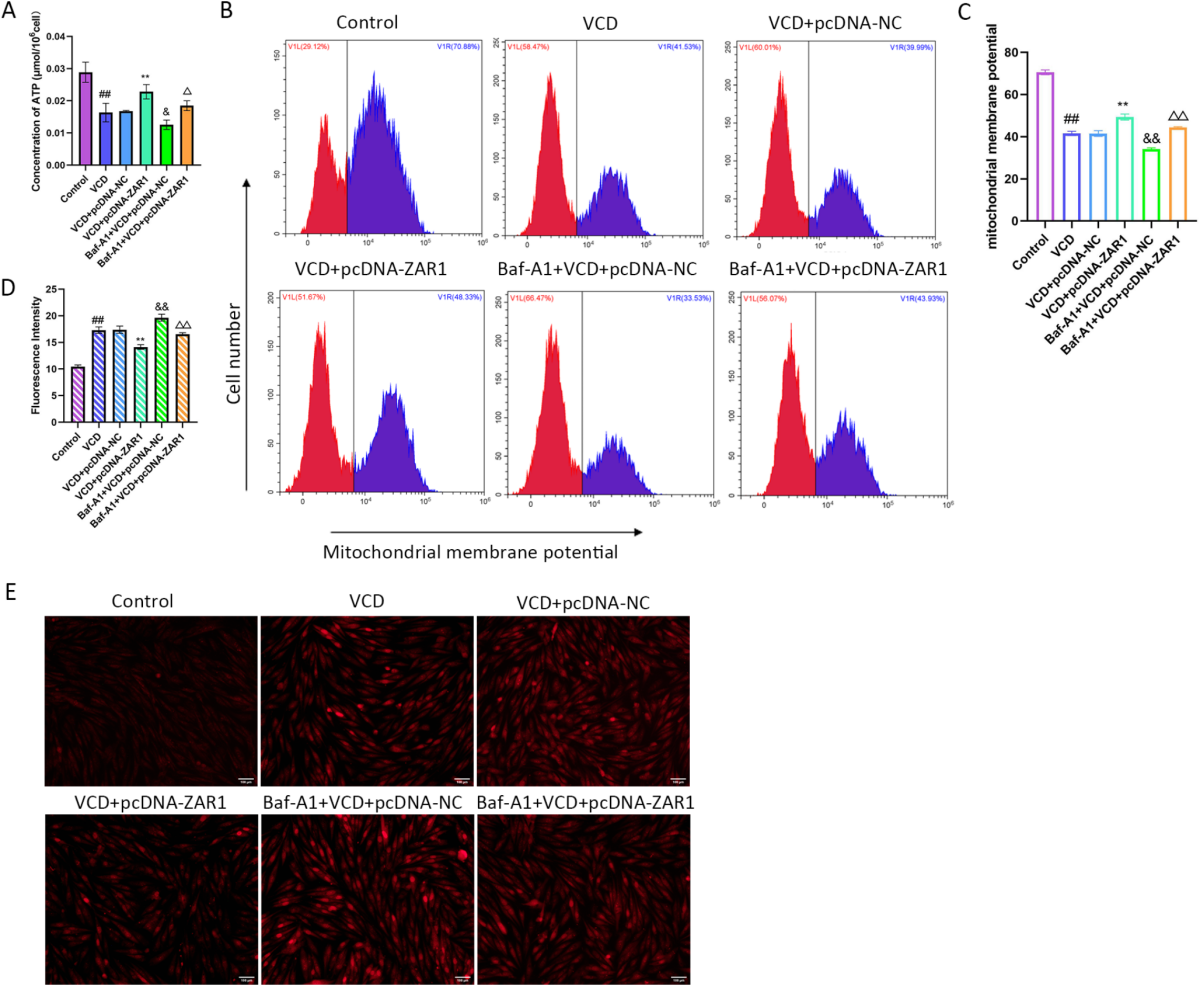
Baf-A1 reversed the regulation of ZAR1 on mitophagy. **A**. The concentration of ATP after treated with Baf-A1 and ZAR1 overexpression. **B-C** The change of mitochondrial membrane potential after treated with Baf-A1 and ZAR1 overexpression. **D-E**. The level of ROS accumulation after treated with Baf-A1 and ZAR1 overexpression. Scale bar=100 μm. ^#^*P*<0.05, ^##^*P*<0.01 vs. control group, ^*^P<0.05, ^**^P<0.01 vs. VCD+pcDNA-NC group, ^&^*P*<0.05, ^&&^*P*<0.01 vs. VCD+pcDNA-NC group, ^△^*P*<0.05, ^△△^*P*<0.01 vs. VCD+pcDNA-ZAR1group

As shown in Fig. 5A, although ZAR1 elevated the level of ATP, the Baf-A1 treatment caused notably reduced concentration of ATP again. The flow cytometry analysis presented that mitochondrial membrane potential was elevated after ZAR1 overexpression while it was significantly declined after combined Baf-A1 treatment (Fig. 5B, C). Besides, the decreased ROS accumulation led by ZAR1 was also reversed by Baf-A1 (Fig. 5D, E). These results indicated that the protective effect of ZAR1 can be inhibited by autophagy inhibitor.

### Decreased Expression of ZAR1 and Increased Levels of Apoptosis and Oxidative Stress in POI Mice

**Fig. 6:**
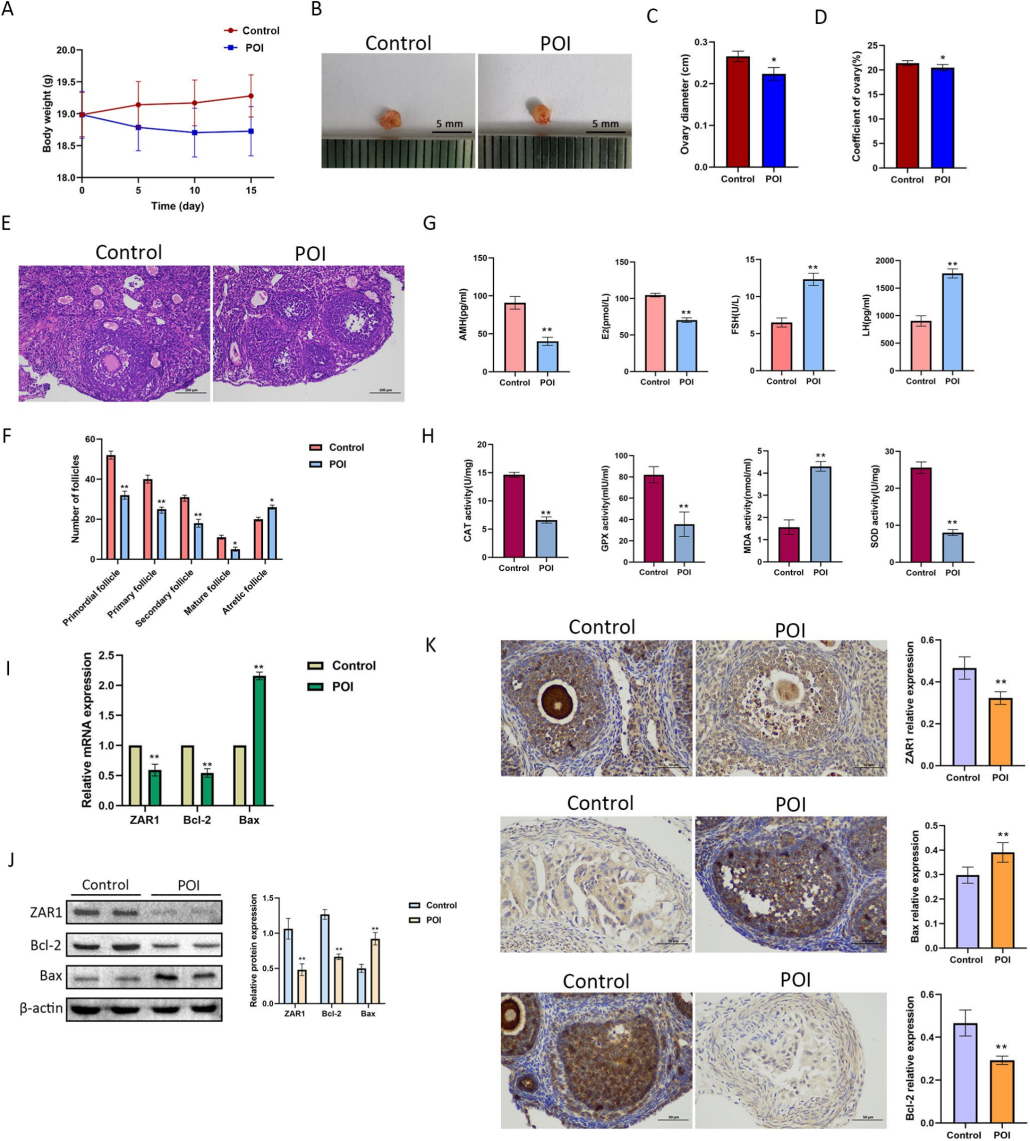
ZAR1 expression was decreased in POI mice. **A**. The body weight of each group (*n*=15). **B**. The morphology of the ovary. **C-D**. The ovarian diameter and ovarian index of each group (*n*=15). **E-F**. Representative images of **H&E** staining of ovarian tissues, and follicle count in each group. Scale bar=100 μm. **G**. The level of steroid hormones in each group. **H**. The level of CAT, GPX, MDA, and SOD in each group (*n*=15). **I**. The mRNA expression of Bax, Bcl-2, and ZAR1 in each group. **J**. The protein expression of Bax, Bcl-2, and ZAR1 in each group. **K**. Representative images of immunohistochemistry detection on Bax, Bcl-2, and ZAR1 in each group. Scale bar=50 μm. *P<0.05, **P<0.01

The POI mice models were built by VCD. Compared with the normal mice, the POI mice had smaller ovarian diameter and index (Fig. 6B-D). The number of primordial follicles, primary follicles, secondary follicles and mature follicles was notably declined while the number of atretic follicles was increased in POI mice (Fig. 6E, F). Compared with the control group, POI mice had lower levels of E2 and AMH and higher level of FSH and LH (Figure 6G). The oxidative stress injury was more obvious in the POI mice for the increased level of MDA and reduced level of CAT, GPX and SOD (Fig. 6H). Meanwhile, the mRNA and protein expressions of *ZAR1* and *Bcl-2* was down-regulated while *Bax* had the opposite tendency (Fig. 6I-K). These results indicated that the mice with less expression of ZAR1 had more serious ovarian damages.

## Discussion

Primary ovarian insufficiency (POI) is a significant reproductive disorder affecting women of child-bearing age. Besides infertility, the disorder of reproductive endocrinology and psychological injury also lead to many adverse effects on women who suffer from it. A great number of researches showed that POI may be related to genetic variation, autoimmune disease, radiation and chemotherapy, surgery caused iatrogenic injury and spontaneous presentation [4, 11, 28, 29]. Among various pathogenic factors, the genetic variation is coming into more and more focus for its diversity and broad effects. Therefore, further research is essential to explore the mechanisms and targets involved, aiming for more precise treatment.

In this study, we found the expression of ZAR1 was reduced in women with POI, and its expression level was negatively related to the FSH level. Previous literature also reported similar findings, which are consistent with the trend of this study [30]. This suggests that ZAR1 may play a protective role in ovarian function, and its downregulation could contribute to ovarian aging and POI. The lower FSH levels observed in our POI patients may reflect early-stage ovarian dysfunction or variability in the patient cohort.

The VCD-induced POI model has been reported in previous experimental literature to show a good induction effect [31, 32]. In the POI model, VCD treatment induced ovarian damage characterized by smaller ovarian diameter and index, decreased numbers of primordial, primary, secondary, and mature follicles, and increased numbers of atretic follicles. POI mice also exhibited elevated FSH and LH levels, decreased E2 and AMH levels, and increased oxidative stress markers (MDA). These findings confirm the effectiveness of the VCD model in inducing POI. However, the modest phenotype of the VCD model suggests that additional models (e.g., androgen receptor deficiency or autoimmune models) may be needed to better understand the mechanisms underlying POI.

Ovarian granulosa cells play a vital role in producing sex hormones necessary for maintaining women’s normal reproductive function, such as oogenesis, folliculogenesis, ovulation, and regulation of the menstrual cycle [33, 34]. We investigated the functional role of ZAR1 in ovarian granulosa cells using the KGN cell line. Our results showed that ZAR1 overexpression significantly inhibited apoptosis and cell cycle arrest in VCD-treated KGN cells. This was evidenced by increased cell viability, reduced apoptosis levels, and altered expression of apoptosis-related proteins (Bax, Bcl-2, and cleaved caspase-3). Additionally, ZAR1 overexpression reversed the cell cycle arrest caused by VCD, increasing the proportion of G0/G1 phase cells and decreasing the proportion of S phase cells. These findings indicate that ZAR1 has anti-apoptotic and cell cycle regulatory effects in granulosa cells, which may contribute to its protective role in ovarian function.

Autophagy is a dynamic process that maintaining the balance of cellular components biosynthesis, recycle and clearance [35]. Both excessive autophagy and deficient autophagy can harm normal ovarian function and cause a decline in reproductive capacity [36, 37]. LC3 II is the membrane marker for autophagosomes and is involved in the process of autophagosome formation and elongation [38]. P62 is an autophagy-related factor that can be degraded by proteolytic enzymes, and its accumulation indicates the arrest of autophagy [39]. Mitochondria are responsible for the energy support of germ cells to maintain steroid hormone synthesis, calcium regulation and other important cellular processes [40]. Hence, protecting mitochondrial quality has important implications for maintaining reproductive health. Mitophagy is a special form of autophagy involved in the degradation and clearance of damaged mitochondria [41]. In this study, VCD treatment caused decreased concentration of ATP and mitochondrial membrane potential, leading to mitochondrial dysfunction, but ZAR1 overexpression restored the damaged mitochondrial function. VCD treatment increased the expression of LC3 II/I, decreased the expression of p62, and activated autophagy flux. However, ZAR1 overexpression reversed these phenomena, indicating that ZAR1 may protect ovarian granulosa cells via inhibiting excessive mitophagy.

Furthermore, we found that the rescue effects of ZAR1 in KGN cells could be blocked by the autophagy inhibitor Baf-A1, suggesting that ZAR1 may be regulated by autophagy and confirming the importance of the interaction between ZAR1 and autophagy-related factors in the development of POI. The use of the autophagy inhibitor Baf-A1 provided further insights into the regulatory mechanisms of ZAR1. However, Baf-A1 is a general autophagy inhibitor that can also promote apoptosis, making it difficult to determine whether the observed effects are directly linked to ZAR1’s interaction with autophagy or a direct effect of Baf-A1 on apoptosis. Future studies using ZAR1-specific inhibitors and additional autophagy markers are needed to clarify this.

Given that oxidative stress can be mediated by mitochondria, we also focused on the changes in oxidative stress level. Oxidative stress is defined as the imbalance between oxidants and antioxidants, leading to suspension of redox signaling and molecular damage [42]. The scavenging activity of oxidizing agents in the ovary dramatically declines with age [43]. Our results showed that ZAR1 overexpression reduced the ROS accumulation caused by VCD in KGN cells. Meanwhile, the levels of antioxidant enzymes were decreased, while the levels of reactive oxygen species product were increased in ZAR1 low-expressed mice. Overall, these findings suggest that ZAR1 alleviate oxidative stress injury in the ovary and thus delay the ovarian aging.

The PI3K/Akt signaling pathway is a key regulator of cell survival, proliferation, and metabolism. It has been shown to play a crucial role in ovarian follicular development and oocyte quality [44, 45]. ZAR1 has been reported to interact with the PI3K/Akt pathway, although the specific mechanisms remain unclear. ZAR1 may regulate apoptosis and autophagy in granulosa cells through the PI3K/Akt pathway. Future research should focus on elucidating the direct interactions between ZAR1 and components of the PI3K/Akt pathway, as well as other signaling pathways involved in ovarian function.

Although our study confirmed the importance of ZAR1 in ovarian injury, some limitations need to be pointed out. First, we only conducted a preliminary exploration of ZAR1 in the ovary, and deeper regulatory mechanisms need to be studied. In addition, we focused on a single molecule, but the occurrence and development of diseases are the result of the intercoordination of multiple molecules. Whether ZAR1 can interact with other molecules is unknown.

## Conclusion

This study demonstrates that ZAR1 inhibits apoptosis, cell cycle arrest, mitophagy and oxidative stress in the ovary, thereby alleviating the ovarian damages. These results suggest that ZAR1 is a potential target for the precaution and treatment of ovarian aging and related ovarian damages.

## Electronic Supplementary Material

Below is the link to the electronic supplementary material.

Supplementary Material 1

## Data Availability

The datasets used and/or analysed during the current study are available from the corresponding author on reasonable request.
