# Supplementary material for: The Regulation of ZAR1 on Apoptosis and Mitophagy in Ovarian Granular Cells and Primary Ovarian Insufficiency (POI) Mice
Source: Reprod Sci. 2025 Apr 11;32(10):3429–41. doi: 10.1007/s43032-025-01857-z (PMC12546325; doi:10.1007/s43032-025-01857-z)
Supplement: Supplementary file 1 — Supplementary Material 1 [file 43032_2025_1857_MOESM1_ESM.docx]

supplementary materials

Figure 2A

| Target | Image |
| --- | --- |
| ꞵ-actin | 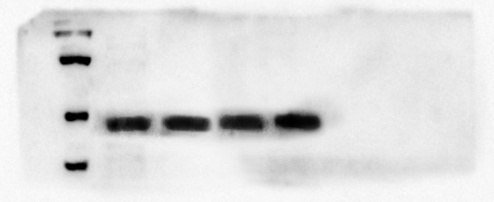 |
|  | 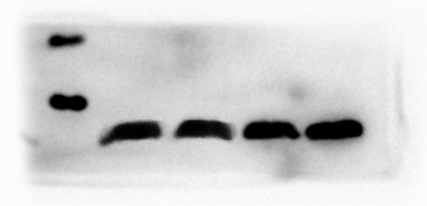 |
|  | 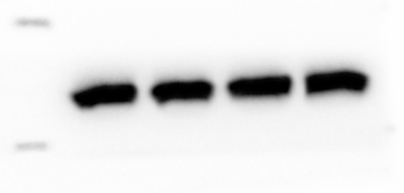 |
| ZAR1 | 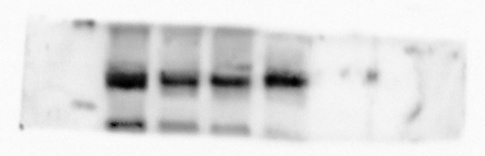 |
|  | 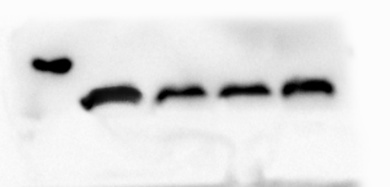 |
|  | 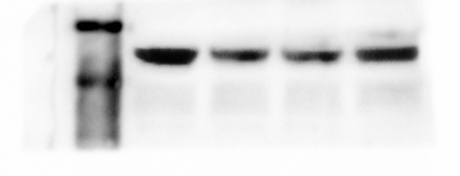 |

Figure 2F

| Target | Image |
| --- | --- |
| Bax | 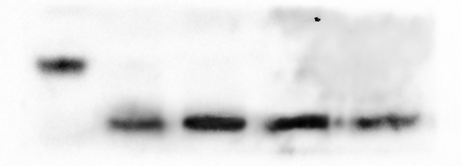 |
|  | 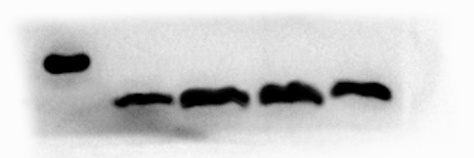 |
|  | 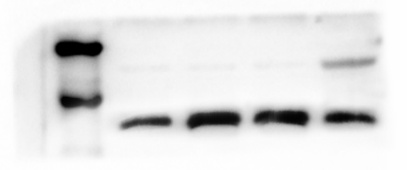 |
| Bcl-2 | 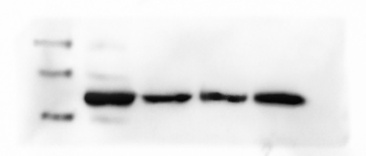 |
|  | 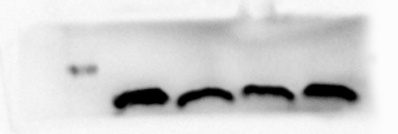 |
|  | 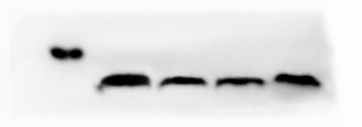 |
| Cleaved caspse-3 | 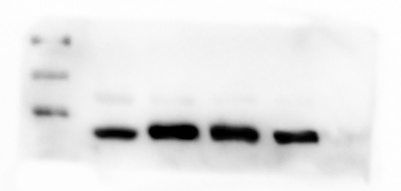 |
|  | 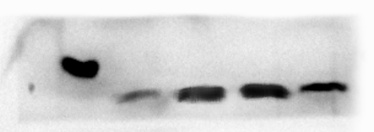 |
|  | 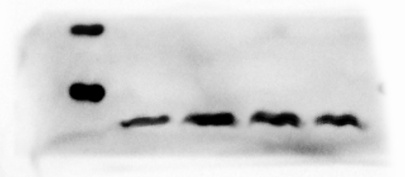 |

Figure 2J

| Target | Image |
| --- | --- |
| Cyclin C | 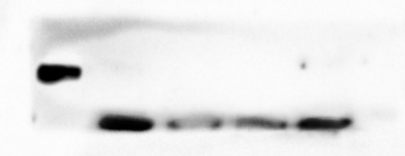 |
|  | 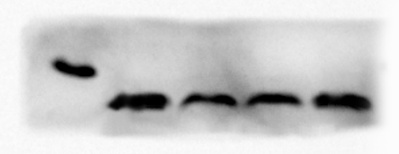 |
|  | 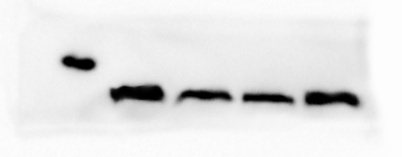 |
| Cyclin D1 | 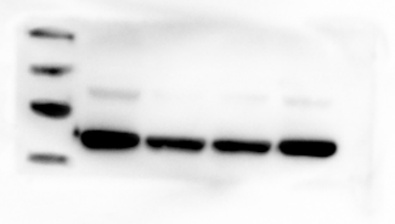 |
|  | 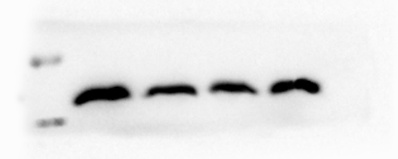 |
|  | 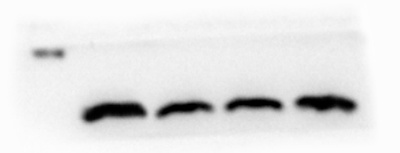 |
| Cyclin E | 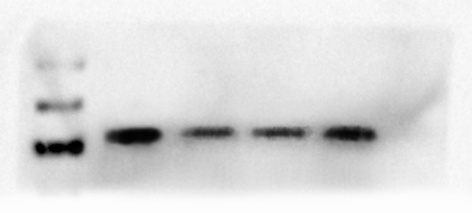 |
|  | 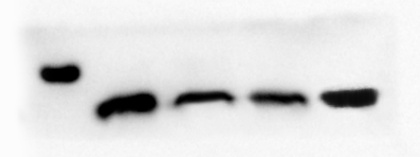 |
|  | 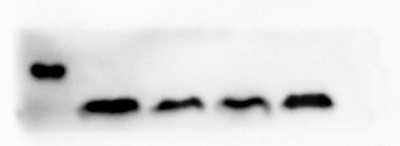 |
| CDK2 | 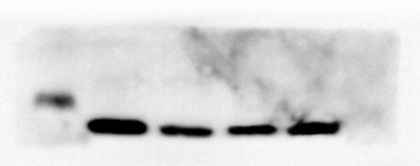 |
|  | 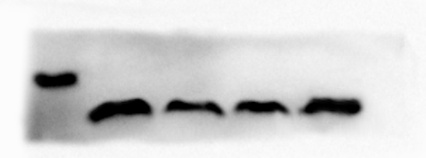 |
|  | 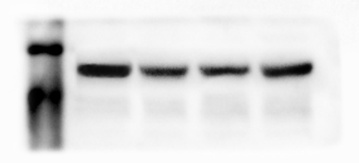 |

Figure 3F

| Target | Image |
| --- | --- |
| LC3 | 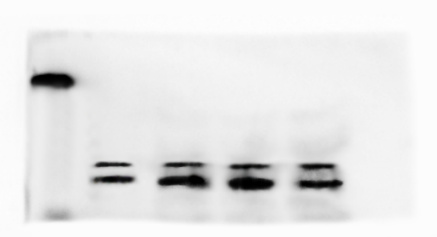 |
|  | 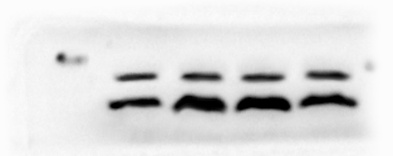 |
|  | 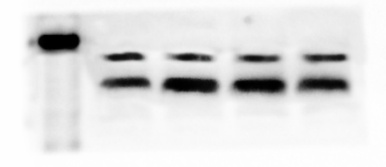 |
| P62 | 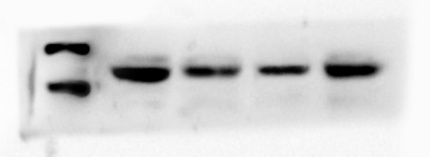 |
|  | 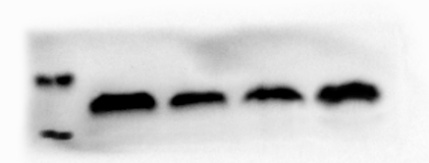 |
|  | 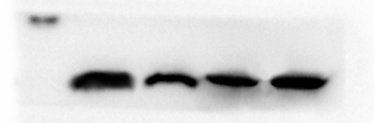 |

Figure 4D

| Target | Image |
| --- | --- |
| ꞵ-actin | 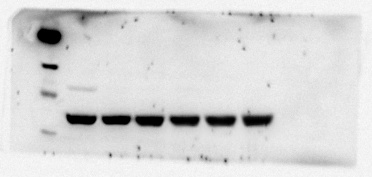 |
|  | 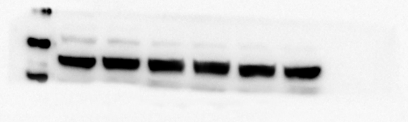 |
|  | 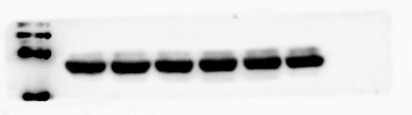 |
| Bax | 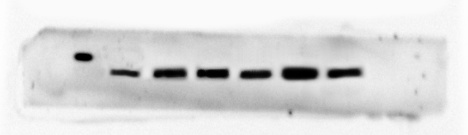 |
|  | 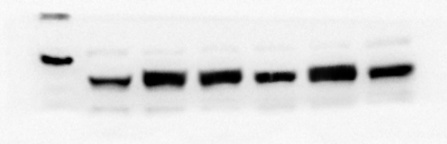 |
|  | 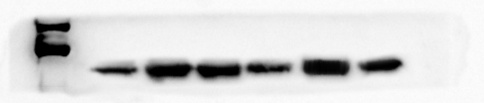 |
| Bcl-2 | 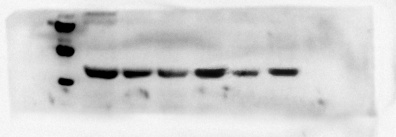 |
|  | 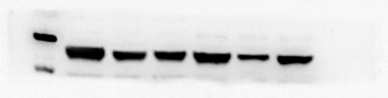 |
|  | 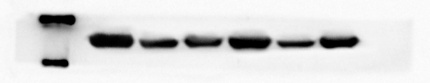 |
| Cleaved caspase-3 | 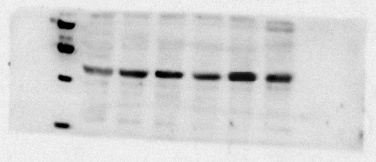 |
|  | 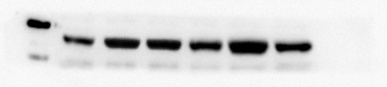 |
|  | 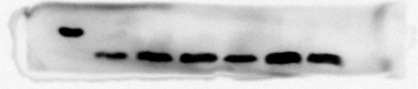 |

Figure 4H

| Target | Image |
| --- | --- |
| Cyclin C | 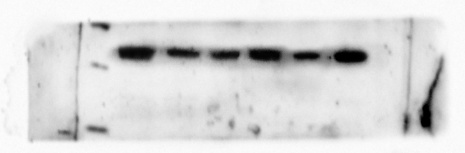 |
|  | 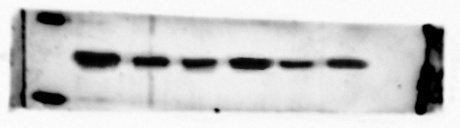 |
|  | 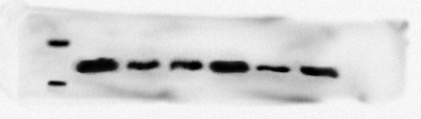 |
| Cyclin D1 | 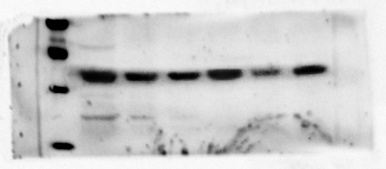 |
|  | 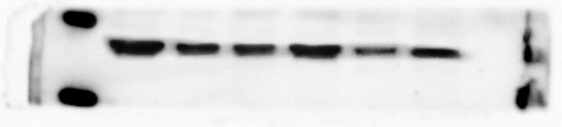 |
|  | 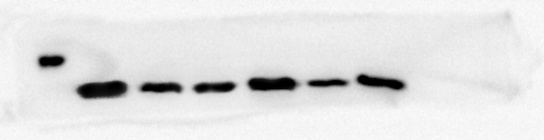 |
| Cyclin E | 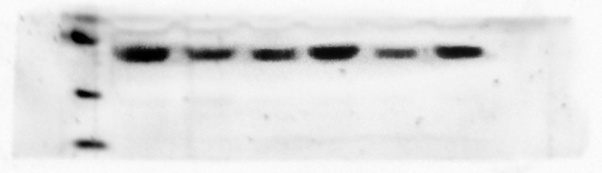 |
|  | 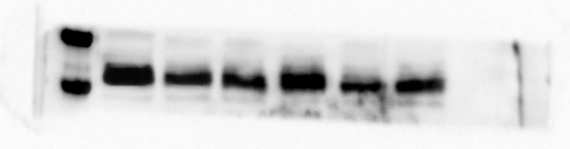 |
|  | 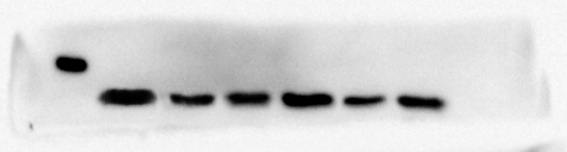 |
| CDK2 | 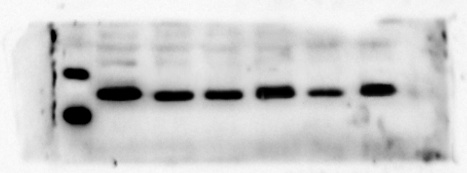 |
|  | 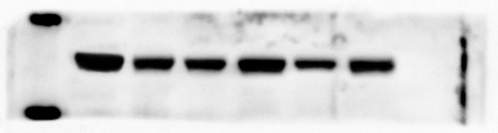 |
|  | 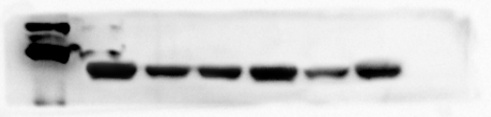 |

Figure 6G

| Target | Image |
| --- | --- |
| ꞵ-actin | 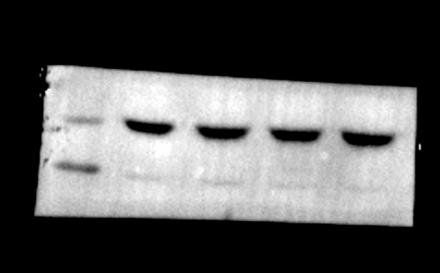 |
|  | 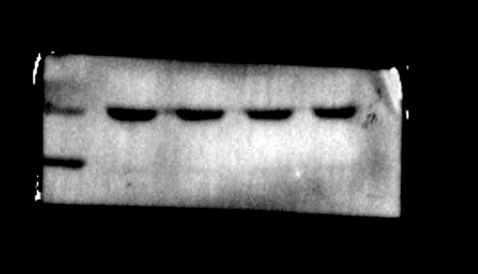 |
|  | 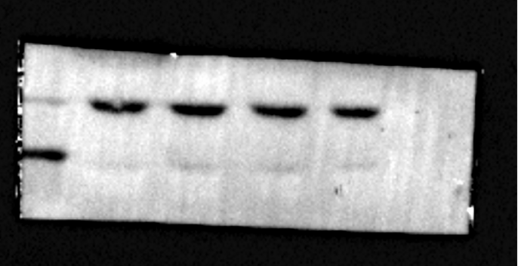 |
|  | 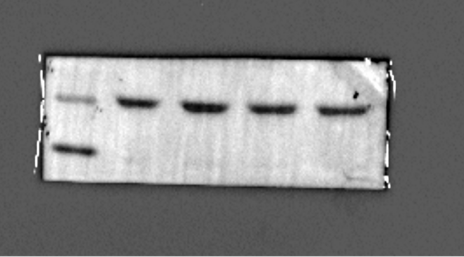 |
|  | 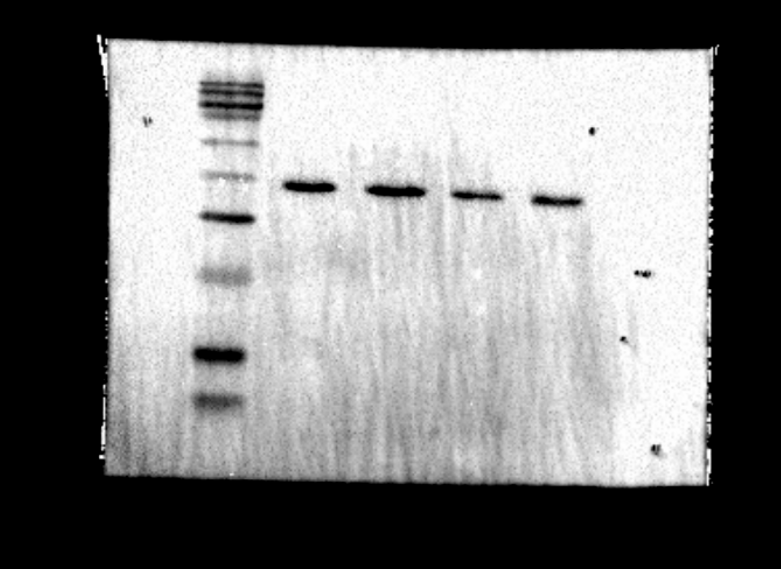 |
| ZAR1 | 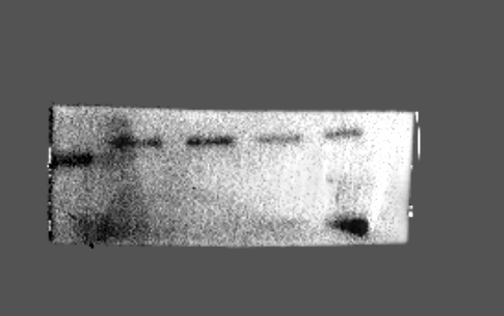 |
|  | 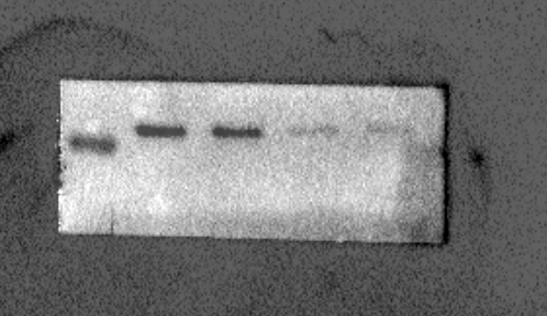 |
|  | 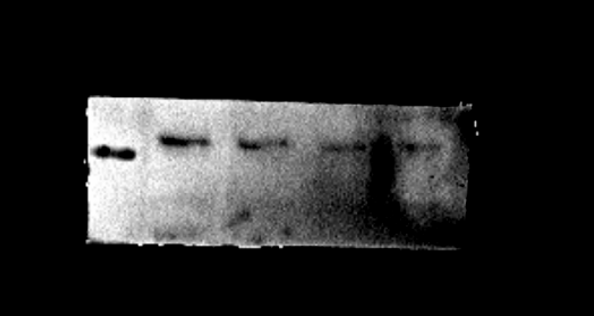 |
|  | 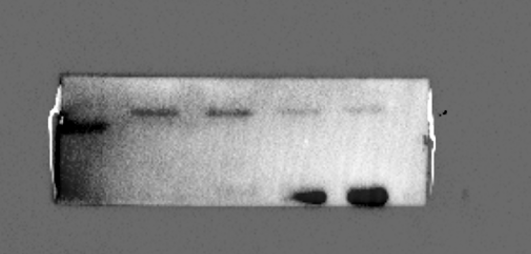 |
|  | 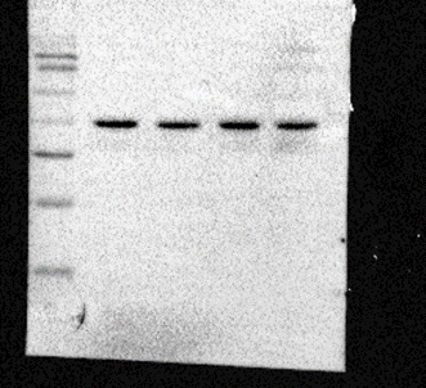 |
| Bax | 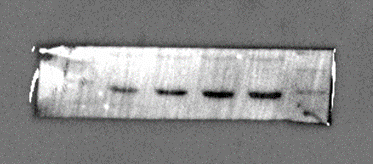 |
|  | 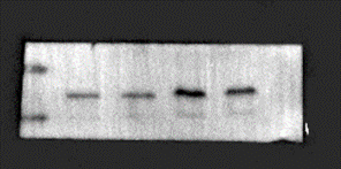 |
|  | 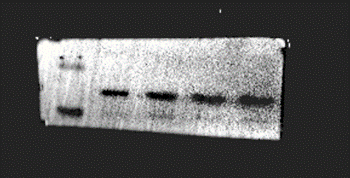 |
|  | 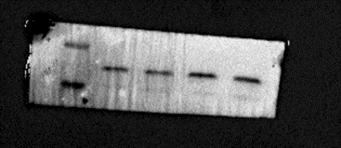 |
|  | 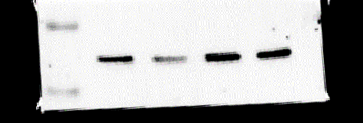 |
| Bcl-2 | 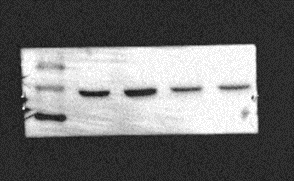 |
|  | 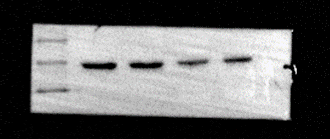 |
|  | 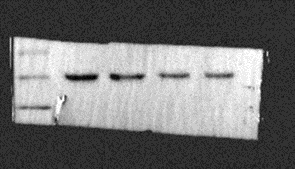 |
|  | 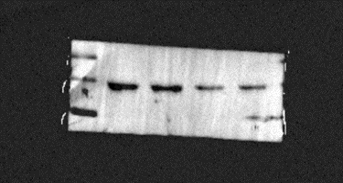 |
|  | 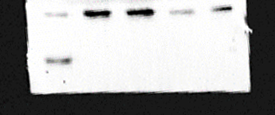 |
